# Synthesis and crystal structure of a two-dimensional Co^II^ coordination polymer: poly[(μ_3_-3-carb­oxy­benzoato)[μ_2_-5-(pyridin-4-yl)-1*H*,2′*H*-3,3′-bi[1,2,4-triazole]]cobalt(II)]

**DOI:** 10.1107/S205698901701533X

**Published:** 2017-10-27

**Authors:** Chao-Jun Du, Xiao-Na Zhao, Bao-Yong Chen

**Affiliations:** a Department of Biology and Chemical Engineering, Nanyang Institute of Technology, 473004 Nanyang, Henan, People’s Republic of China; b Department of Chemical and Chemical Engineering, Guang Xi University, 530000 Nanning, Guangxi, People’s Republic of China; c Laiwu Steel Hospital, 271126 Laiwu, Shandong, People’s Republic of China

**Keywords:** crystal structure, cobalt complex, 5-(pyridin-4-yl)-1*H*,2′*H*-3,3′-bi(1,2,4-triazole), benzene-1,3-di­carb­oxy­lic acid, octa­hedral geometry, hydrogen bond

## Abstract

In the title compound, [Co(C_8_H_5_O_4_)(C_9_H_6_N_7_)]_*n*_, the divalent Co^II^ atom is six-coordinated to three N atoms from two symmetrical 5-(pyridin-4-yl)-1*H*,2′*H*-3,3′-bi[1,2,4-triazole] (H_2_pyttz) ligands and three O atoms from three symmetrical 3-carb­oxy­benzoate (Hbdic) ligands, leading to a distorted {CoN_3_O_3_} octa­hedral coordination environment. Two Co^II^ cations are linked by four bridging carboxyl­ate groups to generate a dinuclear [Co_2_(CO_2_)_4_] unit. The dinuclear units are further connected into a chain along [010] *via* the Hbdic ligands. The other infinite chain, along [100], is formed through the H_2_pyttz ligands. Finally, the two kinds of chains are cross-linked, by sharing the Co^II^ cations, into a two-dimensional network. In the crystal, adjacent layers are further linked by O—H⋯N hydrogen bonds into a three-dimensional framework.

## Abstract 2

The divalent Co^II^ atom is six-coordinated by three N atoms from two symmetrical 5-(pyridin-4-yl)-1*H*,2′*H*-3,3′-bi[1,2,4-triazole] (H_2_pyttz) ligands and three O atoms from three symmetrical 3-carb­oxy­benzoate (Hbdic) ligands, leading to a distorted {CoN_3_O_3_} octa­hedral coordination environment. Two Co^II^ cations are linked by four bridging carboxyl­ate groups to generate a dinuclear [Co_2_(CO_2_)_4_] unit.

## Chemical context   

In recent years, the design and synthesis of coordination polymers (CPs) or metal–organic frameworks (MOFs) have attracted great inter­est because of their fascinating architectures and potential applications in areas such as gas storage and separation, catalysis, fluorescence, magnetism, mol­ecular recognition, conductivity *etc* (Kitagawa *et al.*, 2004 ▸; Zhou *et al.*, 2012 ▸; Cavka *et al.*, 2014 ▸; Zhang *et al.*, 2014 ▸; Huang *et al.*, 2017 ▸; Nath *et al.*, 2016 ▸; Ni *et al.*, 2017 ▸; Yi *et al.*, 2016 ▸; Sun *et al.*, 2016 ▸). It is well known that organic ligands play a crucial role in the rational design and synthesis of coordination polymers (Li & Sato, 2017 ▸; Sun & Sun, 2015 ▸). Among the many organo­nitro­gen ligands, the rigid 5-(pyridin-4-yl)-1*H*,2′*H*-3,3′-bi(1,2,4-triazole) ligand (H_2_pyttz) attracted our attention for the following reasons. First, the H_2_pyttz ligand possesses seven potential N-donor coordination sites and can exhibit various coordination modes. Second, the uncoordinated N atoms are helpful for the construction of hydrogen bonds. The hydrogen bonds not only increase the diversity of coordination polymer structures, but also enhance their stability. With an increasing inter­est in H_2_pyttz organometallic systems, we report herein on the synthesis and crystal structure of the title compound [Co(C_8_H_5_O_4_)(C_9_H_6_N_7_)]_*n*_, (I).
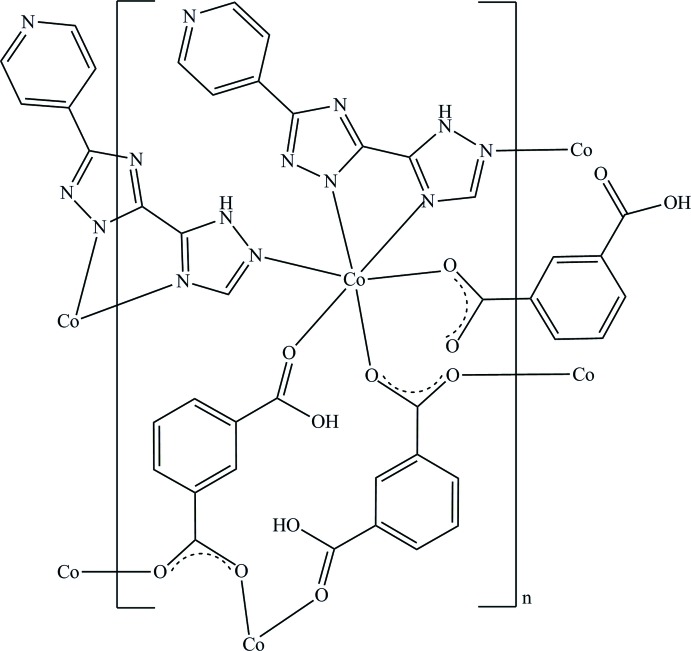


## Structural commentary   

**Figure 1:**
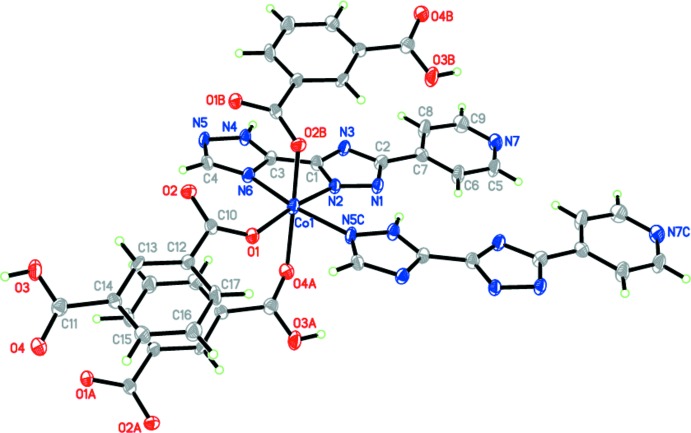
Coordination environment of the Co^II^ cation in (I) showing the atomic numbering scheme, with displacement ellipsoids drawn at the 50% probability level. [Symmetry codes: (*A*) 1 − *x*, −*y*, 1 − *z*; (*B*) 1 − *x*, 1 − *y*, 1 − *z*; (*C*) −1 + *x*, *y*, *z*.]

The asymmetric unit of (I) contains one independent Co^II^ cation, one partially deprotonated Hpyttz^−^ ligand and one partial deprotonated Hbtc^−^ ligand. Notably, the deprotonated Hbtc^−^ ligand adopts two different coordination modes. The deprotonated carboxyl­ate group has a bis­(monodentate) coordination mode to bridge two Co^II^ centers while the undeprotonated carb­oxy­lic group adopts a monodentate mode. As shown in Fig. 1 ▸, the Co^II^ cation is six-coordinated to three carb­oxy­lic oxygen atoms from three symmetrical Hbtc^−^ ligands and three nitro­gen atoms from two symmetrical Hpyttz^−^ ligands in a distorted [CoN_3_O_3_] octa­hedral coordination geometry. Four bridging carboxyl­ate groups link two Co^II^ cations to generate a dinuclear [Co_2_(CO_2_)_4_] unit, which is further connected into an infinite chain along the *b-*axis direction. There exist eight- and 16-membered metallamacrocycles in the chain structure. In the 16-membered metallamacrocycle, the dihedral angle between the two aromatic rings is 0°, indicating the parallel orientation of the two aromatic rings.

The other infinite linear chain is along the *a-*axis direction with a Co⋯Co distance of 6.5825 (5) Å and Co—Co—Co angle of 180.00° and it is also generated through the coordination between the Hpyttz^−^ ligands and the Co^II^ cations. In the complex, the Hpyttz^−^ ligand is almost coplanar, with dihedral angles of 7.48 (4), 6.87 (4) and 4.43 (4) ° between the pyridine and the two triazole rings, respectively. Finally, these two kinds of chains are cross-linked, by sharing the Co^II^ cations, into a two-dimensional network.

## Supra­molecular features   

**Figure 2:**
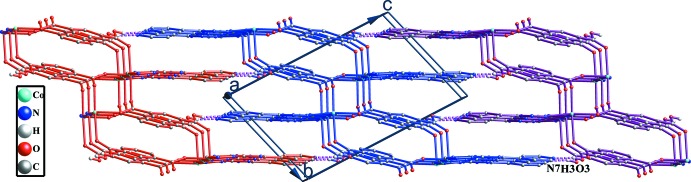
The three-dimensional structure of the title complex formed by the O—H⋯N hydrogen bonds (dashed lines) between adjacent networks (depicted in different colours). H atoms not involved in hydrogen bonds have been omitted for clarity.

In the crystal, adjacent two-dimensional networks are packed parallel to each other in an ⋯*AAAA*⋯ fashion (Fig. 2 ▸). It should be noted that the carb­oxy­lic oxygen atom O3 and the uncoordinated nitro­gen atom N7 in adjacent networks inter­act with each other and form strong O3—H3⋯N7 hydrogen bonds (Table 1 ▸), which further link the two-dimensional networks into a three-dimensional supra­molecular architecture.

## Database survey   

A search of the Cambridge Structural Database (Version 5.37; Groom *et al.*, 2016 ▸) for 5-(pyridin-4-yl)-1*H*,2′*H*-3,3′-bi(1,2,4-triazole) reveals five structures. Of these, there is only one Co^II^ coordination structure (ZOTDIX; Gong *et al.*, 2014 ▸). In this structure, the pyridyl nitro­gen atom is not coordinated to the Co^II^ cation.

## Synthesis and crystallization   

A mixture of Co(NO_3_)_2_^.^6H_2_O (0.10 mmol), 5-(pyridin-4-yl)-1*H*,2′*H*-3,3′-bi(1,2,4-triazole) (0.10 mmol), benzene-1,3-di­carb­oxy­lic acid (0.10 mmol) and H_2_O (10 ml) was stirred at room temperature for 30 min. When the pH value had been adjusted to about 7.0 with 0.1 *M* NaOH, the mixture was sealed in a 20 ml Tefon-lined stainless-steel reactor and then heated to 433 K for 72 h under autogenous pressure, and then slowly cooled to room temperature at a rate of 5 K h^−1^. Pink block-shaped crystals of the title complex were isolated, washed with distilled water, and dried in air (yield 52%).

## Refinement   

Crystal data, data collection and structure refinement details are summarized in Table 2 ▸. H atoms attached to C and N atoms were placed in calculated positions (C—H = 0.93 Å, N—H = 0.86 Å) and refined as riding atoms with *U*_iso_(H) = 1.2*U*_eq_(C,N), respectively. The carboxyl H atom was located in the difference Fourier-map and refined isotropically with *U*_iso_(H) = 1.5*U*_eq_(O).

## Supplementary Material

Crystal structure: contains datablock(s) global, I. DOI: 10.1107/S205698901701533X/kq2016sup1.cif

Structure factors: contains datablock(s) I. DOI: 10.1107/S205698901701533X/kq2016Isup2.hkl

CCDC reference: 1498228

Additional supporting information:  crystallographic information; 3D view; checkCIF report

## Figures and Tables

**Table 1:** Hydrogen-bond geometry (Å, °)

*D*—H⋯*A*	*D*—H	H⋯*A*	*D*⋯*A*	*D*—H⋯*A*
O3—H3⋯N7^i^	0.89 (5)	1.72 (5)	2.592 (4)	166 (5)

Symmetry code: (i) 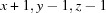.

**Table 2:** Experimental details

Crystal data	
Chemical formula	[Co(C_8_H_5_O_4_)(C_9_H_6_N_7_)]
*M*_r_	436.26
Crystal system, space group	Triclinic, *P* 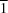
Temperature (K)	293
*a*, *b*, *c* (Å)	6.5825 (5), 9.0574 (12), 13.9842 (12)
α, β, γ (°)	74.214 (1), 84.690 (2), 82.303 (1)
*V* (Å^3^)	793.69 (14)
*Z*	2
Radiation type	Mo *K*α
μ (mm^−1^)	1.13
Crystal size (mm)	0.20 × 0.18 × 0.15

Data collection	
Diffractometer	Bruker SMART CCD area detector
Absorption correction	Multi-scan (*SADABS*; Bruker, 2008 ▸)
*T*_min_, *T*_max_	0.797, 0.858
No. of measured, independent and observed [*I* > 2σ(*I*)] reflections	5565, 3507, 2338
*R*_int_	0.055
(sin θ/λ)_max_ (Å^−1^)	0.669

Refinement	
*R*[*F* ^2^ > 2σ(*F* ^2^)], *wR*(*F* ^2^), *S*	0.061, 0.113, 1.09
No. of reflections	3507
No. of parameters	265
H-atom treatment	H atoms treated by a mixture of independent and constrained refinement
Δρ_max_, Δρ_min_ (e Å^−3^)	0.49, −0.59

Computer programs: *SMART* and *SAINT* (Bruker, 2008 ▸), *SHELXT* (Sheldrick, 2015*a* ▸), *SHELXL2014* (Sheldrick, 2015*b* ▸), *SHELXTL* (Sheldrick, 2008 ▸) and *DIAMOND* (Brandenburg & Putz, 2006 ▸).

## supplementary crystallographic information

### Crystal data

**Table:**

[Co(C_8_H_5_O_4_)(C_9_H_6_N_7_)]	*Z* = 2
*M**_r_* = 436.26	*F*(000) = 442
Triclinic, *P*1	*D*_x_ = 1.825 Mg m^−^^3^
*a* = 6.5825 (5) Å	Mo *K*α radiation, λ = 0.71073 Å
*b* = 9.0574 (12) Å	Cell parameters from 1163 reflections
*c* = 13.9842 (12) Å	θ = 3.0–28.3°
α = 74.214 (1)°	µ = 1.13 mm^−^^1^
β = 84.690 (2)°	*T* = 293 K
γ = 82.303 (1)°	Block, pink
*V* = 793.69 (14) Å^3^	0.20 × 0.18 × 0.15 mm

### Data collection

**Table:**

Bruker SMART CCD area detector diffractometer	2338 reflections with *I* > 2σ(*I*)
Radiation source: fine-focus sealed tube	*R*_int_ = 0.055
φ and ω scans	θ_max_ = 28.4°, θ_min_ = 3.0°
Absorption correction: multi-scan (SADABS; Bruker, 2008)	*h* = −8→8
*T*_min_ = 0.797, *T*_max_ = 0.858	*k* = −11→12
5565 measured reflections	*l* = −16→18
3507 independent reflections	

### Refinement

**Table:**

Refinement on *F*^2^	Primary atom site location: difference Fourier map
Least-squares matrix: full	Secondary atom site location: difference Fourier map
*R*[*F*^2^ > 2σ(*F*^2^)] = 0.061	Hydrogen site location: mixed
*wR*(*F*^2^) = 0.113	H atoms treated by a mixture of independent and constrained refinement
*S* = 1.09	*w* = 1/[σ^2^(*F*_o_^2^) + (0.0172*P*)^2^ + 0.0319*P*] where *P* = (*F*_o_^2^ + 2*F*_c_^2^)/3
3507 reflections	(Δ/σ)_max_ < 0.001
265 parameters	Δρ_max_ = 0.49 e Å^−^^3^
0 restraints	Δρ_min_ = −0.59 e Å^−^^3^

### Special details

**Table:**

Geometry. All esds (except the esd in the dihedral angle between two l.s. planes) are estimated using the full covariance matrix. The cell esds are taken into account individually in the estimation of esds in distances, angles and torsion angles; correlations between esds in cell parameters are only used when they are defined by crystal symmetry. An approximate (isotropic) treatment of cell esds is used for estimating esds involving l.s. planes.

### Fractional atomic coordinates and isotropic or equivalent isotropic displacement parameters (Å^2^)

**Table:**

	*x*	*y*	*z*	*U*_iso_*/*U*_eq_	
Co1	0.36855 (8)	0.42648 (8)	0.65379 (5)	0.02024 (18)	
N1	0.2335 (5)	0.5770 (4)	0.8362 (3)	0.0196 (9)	
N2	0.3746 (5)	0.5162 (4)	0.7767 (3)	0.0182 (9)	
N3	0.5482 (5)	0.6182 (4)	0.8697 (3)	0.0210 (9)	
N4	0.9301 (5)	0.5039 (4)	0.7413 (3)	0.0200 (9)	
H4	0.9832	0.5434	0.7811	0.024*	
N5	1.0369 (5)	0.4445 (4)	0.6689 (3)	0.0197 (9)	
N6	0.7019 (5)	0.4259 (4)	0.6688 (3)	0.0191 (9)	
N7	0.0562 (5)	0.8553 (5)	1.1093 (3)	0.0273 (10)	
O1	0.3534 (4)	0.3140 (4)	0.5493 (2)	0.0209 (7)	
O2	0.6377 (4)	0.3585 (4)	0.4500 (2)	0.0209 (7)	
O3	0.8393 (5)	−0.0128 (4)	0.2340 (3)	0.0342 (10)	
H3	0.917 (7)	−0.071 (6)	0.200 (4)	0.051*	
O4	0.6280 (4)	−0.1888 (4)	0.2391 (2)	0.0257 (8)	
C1	0.5576 (6)	0.5438 (5)	0.7995 (3)	0.0180 (10)	
C2	0.3424 (6)	0.6368 (5)	0.8906 (3)	0.0199 (10)	
C3	0.7332 (6)	0.4911 (5)	0.7405 (3)	0.0173 (10)	
C4	0.8918 (6)	0.3997 (5)	0.6277 (3)	0.0206 (11)	
H4A	0.9183	0.3542	0.5751	0.025*	
C5	−0.0515 (6)	0.7829 (6)	1.0630 (4)	0.0280 (12)	
H5	−0.1909	0.7793	1.0807	0.034*	
C6	0.0347 (6)	0.7140 (6)	0.9909 (4)	0.0282 (12)	
H6	−0.0461	0.6662	0.9598	0.034*	
C7	0.2431 (6)	0.7154 (5)	0.9641 (3)	0.0209 (10)	
C8	0.3536 (6)	0.7892 (6)	1.0130 (4)	0.0297 (12)	
H8	0.4938	0.7923	0.9977	0.036*	
C9	0.2573 (7)	0.8578 (6)	1.0842 (4)	0.0300 (13)	
H9	0.3342	0.9075	1.1159	0.036*	
C10	0.4755 (6)	0.2952 (5)	0.4773 (3)	0.0186 (10)	
C11	0.6668 (6)	−0.0715 (6)	0.2607 (3)	0.0213 (10)	
C12	0.4189 (6)	0.1867 (5)	0.4224 (3)	0.0173 (10)	
C13	0.5649 (6)	0.1212 (5)	0.3625 (3)	0.0191 (10)	
H13	0.6973	0.1499	0.3523	0.023*	
C14	0.5117 (6)	0.0126 (5)	0.3179 (3)	0.0198 (10)	
C15	0.3112 (6)	−0.0227 (5)	0.3286 (3)	0.0235 (11)	
H15	0.2761	−0.0946	0.2982	0.028*	
C16	0.1631 (6)	0.0470 (6)	0.3834 (4)	0.0285 (12)	
H16	0.0276	0.0257	0.3880	0.034*	
C17	0.2189 (6)	0.1497 (6)	0.4316 (3)	0.0239 (11)	
H17	0.1206	0.1943	0.4706	0.029*	

### Atomic displacement parameters (Å^2^)

**Table:**

	*U*^11^	*U*^22^	*U*^33^	*U*^12^	*U*^13^	*U*^23^
Co1	0.0155 (3)	0.0282 (4)	0.0212 (4)	−0.0045 (3)	0.0037 (2)	−0.0142 (3)
N1	0.0152 (18)	0.027 (2)	0.019 (2)	−0.0025 (16)	0.0041 (15)	−0.0124 (19)
N2	0.0152 (17)	0.022 (2)	0.019 (2)	−0.0014 (16)	0.0029 (15)	−0.0101 (19)
N3	0.0155 (18)	0.028 (2)	0.023 (2)	−0.0047 (16)	0.0039 (15)	−0.0132 (19)
N4	0.0158 (18)	0.030 (2)	0.019 (2)	−0.0041 (16)	0.0025 (15)	−0.0150 (19)
N5	0.0120 (17)	0.024 (2)	0.025 (2)	−0.0028 (16)	0.0053 (15)	−0.0112 (19)
N6	0.0159 (17)	0.027 (2)	0.019 (2)	−0.0039 (16)	0.0009 (15)	−0.0129 (19)
N7	0.031 (2)	0.029 (3)	0.025 (2)	−0.0022 (19)	0.0044 (18)	−0.016 (2)
O1	0.0245 (16)	0.0234 (19)	0.0183 (18)	−0.0064 (14)	0.0066 (13)	−0.0120 (15)
O2	0.0201 (15)	0.0205 (18)	0.0247 (19)	−0.0065 (13)	0.0016 (13)	−0.0096 (16)
O3	0.0267 (18)	0.041 (2)	0.043 (2)	−0.0092 (17)	0.0161 (16)	−0.029 (2)
O4	0.0300 (17)	0.026 (2)	0.026 (2)	−0.0057 (15)	0.0039 (14)	−0.0147 (17)
C1	0.018 (2)	0.022 (3)	0.016 (2)	−0.0021 (19)	0.0014 (17)	−0.008 (2)
C2	0.019 (2)	0.025 (3)	0.018 (3)	−0.006 (2)	0.0003 (18)	−0.010 (2)
C3	0.015 (2)	0.020 (3)	0.018 (2)	−0.0010 (18)	0.0004 (17)	−0.008 (2)
C4	0.017 (2)	0.022 (3)	0.026 (3)	0.000 (2)	0.0024 (19)	−0.014 (2)
C5	0.020 (2)	0.038 (3)	0.027 (3)	−0.004 (2)	0.010 (2)	−0.015 (3)
C6	0.021 (2)	0.042 (3)	0.027 (3)	−0.003 (2)	0.006 (2)	−0.021 (3)
C7	0.025 (2)	0.023 (3)	0.017 (3)	0.001 (2)	0.0010 (19)	−0.011 (2)
C8	0.017 (2)	0.044 (3)	0.034 (3)	−0.004 (2)	0.003 (2)	−0.022 (3)
C9	0.031 (3)	0.037 (3)	0.032 (3)	−0.009 (2)	0.002 (2)	−0.024 (3)
C10	0.016 (2)	0.016 (3)	0.023 (3)	0.0015 (19)	0.0000 (18)	−0.004 (2)
C11	0.022 (2)	0.026 (3)	0.016 (3)	−0.001 (2)	−0.0006 (18)	−0.007 (2)
C12	0.019 (2)	0.023 (3)	0.013 (2)	−0.0060 (19)	0.0008 (17)	−0.010 (2)
C13	0.016 (2)	0.025 (3)	0.019 (3)	−0.0109 (19)	0.0068 (17)	−0.009 (2)
C14	0.025 (2)	0.019 (3)	0.017 (2)	−0.004 (2)	0.0040 (18)	−0.008 (2)
C15	0.024 (2)	0.026 (3)	0.026 (3)	−0.008 (2)	0.002 (2)	−0.016 (2)
C16	0.021 (2)	0.038 (3)	0.033 (3)	−0.007 (2)	0.003 (2)	−0.019 (3)
C17	0.025 (2)	0.027 (3)	0.022 (3)	0.002 (2)	0.0048 (19)	−0.013 (2)

### Geometric parameters (Å, º)

**Table:**

Co1—O1	2.012 (3)	O4—C11	1.244 (5)
Co1—O2^i^	2.086 (3)	O4—Co1^iii^	2.262 (4)
Co1—N2	2.097 (3)	C1—C3	1.461 (5)
Co1—N5^ii^	2.162 (3)	C2—C7	1.463 (5)
Co1—N6	2.223 (3)	C4—H4A	0.9300
Co1—O4^iii^	2.262 (4)	C5—C6	1.368 (5)
N1—C2	1.347 (5)	C5—H5	0.9300
N1—N2	1.352 (4)	C6—C7	1.389 (6)
N2—C1	1.345 (5)	C6—H6	0.9300
N3—C1	1.327 (5)	C7—C8	1.384 (6)
N3—C2	1.357 (5)	C8—C9	1.377 (6)
N4—C3	1.318 (5)	C8—H8	0.9300
N4—N5	1.368 (4)	C9—H9	0.9300
N4—H4	0.8600	C10—C12	1.502 (5)
N5—C4	1.320 (5)	C11—C14	1.495 (5)
N5—Co1^iv^	2.162 (3)	C12—C17	1.387 (6)
N6—C3	1.338 (5)	C12—C13	1.395 (5)
N6—C4	1.345 (5)	C13—C14	1.394 (5)
N7—C5	1.339 (5)	C13—H13	0.9300
N7—C9	1.339 (5)	C14—C15	1.385 (5)
O1—C10	1.262 (5)	C15—C16	1.377 (5)
O2—C10	1.258 (5)	C15—H15	0.9300
O2—Co1^i^	2.086 (3)	C16—C17	1.391 (6)
O3—C11	1.299 (5)	C16—H16	0.9300
O3—H3	0.89 (5)	C17—H17	0.9300
			
O1—Co1—O2^i^	93.18 (12)	N5—C4—N6	113.9 (4)
O1—Co1—N2	172.28 (15)	N5—C4—H4A	123.1
O2^i^—Co1—N2	94.31 (13)	N6—C4—H4A	123.1
O1—Co1—N5^ii^	87.27 (12)	N7—C5—C6	122.8 (4)
O2^i^—Co1—N5^ii^	91.64 (13)	N7—C5—H5	118.6
N2—Co1—N5^ii^	90.64 (12)	C6—C5—H5	118.6
O1—Co1—N6	105.23 (11)	C5—C6—C7	119.9 (4)
O2^i^—Co1—N6	90.18 (13)	C5—C6—H6	120.1
N2—Co1—N6	76.64 (12)	C7—C6—H6	120.1
N5^ii^—Co1—N6	167.25 (12)	C8—C7—C6	116.9 (4)
O1—Co1—O4^iii^	84.32 (12)	C8—C7—C2	121.6 (4)
O2^i^—Co1—O4^iii^	177.47 (11)	C6—C7—C2	121.4 (4)
N2—Co1—O4^iii^	88.17 (13)	C9—C8—C7	120.4 (4)
N5^ii^—Co1—O4^iii^	87.82 (13)	C9—C8—H8	119.8
N6—Co1—O4^iii^	90.88 (13)	C7—C8—H8	119.8
C2—N1—N2	105.2 (3)	N7—C9—C8	122.0 (4)
C1—N2—N1	105.6 (3)	N7—C9—H9	119.0
C1—N2—Co1	117.7 (3)	C8—C9—H9	119.0
N1—N2—Co1	136.0 (2)	O2—C10—O1	125.2 (4)
C1—N3—C2	100.7 (3)	O2—C10—C12	119.0 (4)
C3—N4—N5	109.5 (3)	O1—C10—C12	115.8 (4)
C3—N4—H4	125.2	O4—C11—O3	123.0 (4)
N5—N4—H4	125.2	O4—C11—C14	121.0 (4)
C4—N5—N4	103.0 (3)	O3—C11—C14	116.1 (4)
C4—N5—Co1^iv^	135.8 (3)	C17—C12—C13	119.2 (4)
N4—N5—Co1^iv^	121.0 (2)	C17—C12—C10	119.8 (4)
C3—N6—C4	103.5 (3)	C13—C12—C10	121.0 (4)
C3—N6—Co1	111.1 (2)	C14—C13—C12	119.8 (4)
C4—N6—Co1	144.9 (3)	C14—C13—H13	120.1
C5—N7—C9	118.1 (4)	C12—C13—H13	120.1
C10—O1—Co1	132.1 (3)	C15—C14—C13	119.8 (4)
C10—O2—Co1^i^	120.5 (3)	C15—C14—C11	118.3 (4)
C11—O3—H3	108 (3)	C13—C14—C11	121.9 (4)
C11—O4—Co1^iii^	124.8 (3)	C16—C15—C14	120.9 (4)
N3—C1—N2	114.7 (3)	C16—C15—H15	119.5
N3—C1—C3	130.7 (4)	C14—C15—H15	119.5
N2—C1—C3	114.6 (3)	C15—C16—C17	119.1 (4)
N1—C2—N3	113.8 (3)	C15—C16—H16	120.5
N1—C2—C7	121.9 (4)	C17—C16—H16	120.5
N3—C2—C7	124.3 (4)	C12—C17—C16	121.1 (4)
N4—C3—N6	110.1 (3)	C12—C17—H17	119.5
N4—C3—C1	130.3 (4)	C16—C17—H17	119.5
N6—C3—C1	119.5 (3)		
			
C2—N1—N2—C1	−0.1 (5)	N1—C2—C7—C8	−173.9 (5)
C2—N1—N2—Co1	−169.6 (4)	N3—C2—C7—C8	5.4 (8)
C3—N4—N5—C4	0.5 (5)	N1—C2—C7—C6	8.8 (7)
C3—N4—N5—Co1^iv^	−174.9 (3)	N3—C2—C7—C6	−171.9 (5)
C2—N3—C1—N2	−0.1 (5)	C6—C7—C8—C9	−0.4 (8)
C2—N3—C1—C3	177.7 (5)	C2—C7—C8—C9	−177.8 (5)
N1—N2—C1—N3	0.1 (5)	C5—N7—C9—C8	0.2 (8)
Co1—N2—C1—N3	171.9 (3)	C7—C8—C9—N7	0.5 (8)
N1—N2—C1—C3	−178.1 (4)	Co1^i^—O2—C10—O1	−88.9 (5)
Co1—N2—C1—C3	−6.3 (5)	Co1^i^—O2—C10—C12	91.8 (4)
N2—N1—C2—N3	0.0 (5)	Co1—O1—C10—O2	−6.3 (7)
N2—N1—C2—C7	179.3 (4)	Co1—O1—C10—C12	173.1 (3)
C1—N3—C2—N1	0.1 (5)	Co1^iii^—O4—C11—O3	104.3 (5)
C1—N3—C2—C7	−179.3 (5)	Co1^iii^—O4—C11—C14	−77.2 (5)
N5—N4—C3—N6	−0.6 (5)	O2—C10—C12—C17	−162.0 (4)
N5—N4—C3—C1	−176.6 (5)	O1—C10—C12—C17	18.6 (6)
C4—N6—C3—N4	0.4 (5)	O2—C10—C12—C13	18.4 (7)
Co1—N6—C3—N4	−173.6 (3)	O1—C10—C12—C13	−161.0 (4)
C4—N6—C3—C1	177.0 (4)	C17—C12—C13—C14	−4.1 (7)
Co1—N6—C3—C1	3.0 (5)	C10—C12—C13—C14	175.5 (4)
N3—C1—C3—N4	−0.2 (9)	C12—C13—C14—C15	3.9 (7)
N2—C1—C3—N4	177.7 (5)	C12—C13—C14—C11	−174.3 (4)
N3—C1—C3—N6	−175.9 (5)	O4—C11—C14—C15	−13.1 (7)
N2—C1—C3—N6	1.9 (6)	O3—C11—C14—C15	165.5 (4)
N4—N5—C4—N6	−0.2 (5)	O4—C11—C14—C13	165.1 (5)
Co1^iv^—N5—C4—N6	174.1 (3)	O3—C11—C14—C13	−16.3 (7)
C3—N6—C4—N5	−0.1 (5)	C13—C14—C15—C16	−0.6 (7)
Co1—N6—C4—N5	170.1 (4)	C11—C14—C15—C16	177.7 (4)
C9—N7—C5—C6	−0.9 (8)	C14—C15—C16—C17	−2.5 (7)
N7—C5—C6—C7	1.0 (8)	C13—C12—C17—C16	1.0 (7)
C5—C6—C7—C8	−0.3 (7)	C10—C12—C17—C16	−178.6 (5)
C5—C6—C7—C2	177.1 (5)	C15—C16—C17—C12	2.3 (8)

Symmetry codes: (i) −*x*+1, −*y*+1, −*z*+1; (ii) *x*−1, *y*, *z*; (iii) −*x*+1, −*y*, −*z*+1; (iv) *x*+1, *y*, *z*.

### Hydrogen-bond geometry (Å, º)

**Table:**

*D*—H···*A*	*D*—H	H···*A*	*D*···*A*	*D*—H···*A*
O3—H3···N7^v^	0.89 (5)	1.72 (5)	2.592 (4)	166 (5)

Symmetry code: (v) *x*+1, *y*−1, *z*−1.
